# Effect of myocardial hemorrhage on functional recovery in patients with reperfused acute myocardial infarction

**DOI:** 10.1186/1532-429X-13-S1-P161

**Published:** 2011-02-02

**Authors:** Yoko Mikami, Andreas Kumar, Matthias G Friedrich

**Affiliations:** 1 Stephenson CMR Centre, Libin Cardiovascular Institute of Alberta, Calgary, AB, Canada; 2 Laval University, The Institute of Cardiology and Pulmonology of Quebec, Quebec, QC, Canada

## Background

Microvascular obstruction (MO) is one of the most important predictors of adverse outcome after reperfusion therapy in acute myocardial infarction (MI). Myocardial hemorrhage has been described as a contributor to reperfusion injury. Due to a lack of appropriate imaging techniques, the consequences of reperfusion hemorrhage have not been studied. The purpose of this study is to investigate the relationship between myocardial hemorrhage and regional left ventricular functional recovery using T2*-weighted cardiovascular magnetic resonance (CMR) imaging.

## Methods

Fourteen patients with reperfused acute MI underwent repeat CMR studies 3 days and 6 months after onset. Studies consisted of function, T2*-weighted, early enhancement (2-5 minutes post contrast) and late gadolinium enhancement (LGE) imaging. On the images acquired 3 days after onset, the presence of hemorrhage was determined on T2*-weighted CMR and the presence of MO was determined on early enhancement CMR. Based on a 16-segment model, each segment was classified into one of 4 groups; (a. no infarction, b. infarction but no microvascular obstruction, c. microvascular obstruction but no hemorrhage, d. microvascular obstruction with hemorrhage). The systolic wall thickening on cine CMR at 6 months was evaluated for regional contractile function.

## Results

When possible results are represented as mean +/- standard deviation. One apical slice was excluded because of the poor image quality. In total, 220 segments were analyzed and 45 segments were positive for infarction. Out of 45 segments with infarction, 18 showed both MO and hemorrhage and 6 showed MO but without hemorrhage. Systolic wall thickening for each group at 6 months was a. 80+/-60%, b. 48+/-34%, c. 41+/-32%, d. 15+/-22%. In the segments with MO but without hemorrhage, systolic wall thickening was not significantly different from those in infarct segments without MO or hemorrhage (41+/-32% vs. 48+/-34%, p=N.S.). In the segments with both MO and hemorrhage, systolic wall thickening was significantly smaller than infarct segments without MO or hemorrhage (15+/-22% vs. 48+/-34%, p<0.05) and those segments with MO but without hemorrhage (15+/-22% vs. 41+/-32%, p<0.05).

## Conclusions

Myocardial hemorrhage as a complication of reperfusion therapy is strongly associated with impaired regional functional recovery at 6 months. The presence of hemorrhage detected on T2*-weighted CMR may be an important predictor for adverse outcome over the presence of MO.

